# Viral genomic DNA packaging machinery

**DOI:** 10.1007/978-3-031-58843-3_9

**Published:** 2024-01-01

**Authors:** Dorothy E.D.P. Hawkins, Owen C. Godwin, Alfred A. Antson

**Affiliations:** 1 York Structural Biology Laboratory, Department of Chemistry, University of York, York YO10 5DD, UK

**Keywords:** DNA translocation, large terminase, small terminase, portal protein, bacteriophage, ATPase, nuclease

## Abstract

Tailed double-stranded DNA bacteriophage employ a protein terminase motor to package their genome into a preformed protein shell - a system shared with eukaryotic dsDNA viruses such as *herpesviruses*. DNA packaging motor proteins represent excellent targets for antiviral therapy, with Letermovir, which binds *Cytomegalovirus* terminase, already licensed as an effective prophylaxis. In the realm of bacterial viruses, these DNA packaging motors comprise three protein constituents: the portal protein, small terminase and large terminase. The portal protein guards the passage of DNA into the preformed protein shell and acts as a protein interaction hub throughout viral assembly. Small terminase recognises the viral DNA and recruits large terminase, which in turn pumps DNA in an ATP dependant manner. Large terminase also cleaves DNA at the termination of packaging. Multiple high-resolution structures of each component have been resolved for different phages, but it is only more recently that the field has moved towards cryo-EM reconstructions of protein complexes. In conjunction with highly informative single particle studies of packaging kinetics, these structures have begun to inspire models for the packaging process and its place among other DNA machines.

## Abbreviations

ASCE: Additional Strand, Conserved Glutamate
ADP: Adenosine diphosphate
ATP: Adenosine triphosphate
ATP-γ-S: Adenosine-5′-o-(3-thio-triphosphate), Tetralithium Salt Bp Base Pair
CMV: *Cytomegalovirus*
CTD: Terminal Domain
Cryo-EM: Cryo-Electron Microscopy
DNA: Deoxyribonucleic Acid dsDNA Double Stranded DNA
EPR: Electron Paramagnetic Resonance
FRET: Fluorescence Resonance Energy Transfer
HTH: Helix Turn Helix
HSV1: *Herpes Simplex Virus 1*
MD: Molecular Dynamics
NTD: N Terminal Domain
RNA: Ribonucleic acid
PDB: Protein Data Bank
SPR: Surface Plasmon Resonance

## Introduction to viral DNA packaging

Encapsulation of genetic material represents an essential process for viral assembly and thus viability. For prokaryotic dsDNA viruses, this process may be fulfilled by one of three distinct DNA packing mechanisms. Bacteriophage with very small genomes (typically less than 20kDa) may utilise the nucleation of capsid proteins around the genomic DNA, in an energy independent manner (Burroughs et al. 2007). However, above this 20kb threshold, (and indeed for most smaller viruses too), bacteriophage genomes encode an ATPase for DNA packaging, which belongs to either Ftsk like or terminase subcategories. FtsK-based motors are utilised exclusively by phage with internal lipid membranes such as *Corticoviruses* and *Tectiviruses*, as well as all *Nucleocytoviricota* eukaryotic viruses such as *Poxvirus* and *Mimivirus* (Burroughs et al. 2007; Chelikani et al. 2014). The FtsK-like motors are also encoded by large eukaryotic transposons known as *Polintons* which may represent the evolutionary origin of viruses with this packaging system (Krupovic and Koonin 2015). Terminase type systems are instead utilised by *Herpesviridae* viruses and tailed bacteriophages (the *Caudivirales*) which are thought to represent up to 96 % of all known phage (Ackermann 2007). The terminase-based motor system has been subject to extensive study and will thus represent the focus of this chapter.

**Figure 1:**
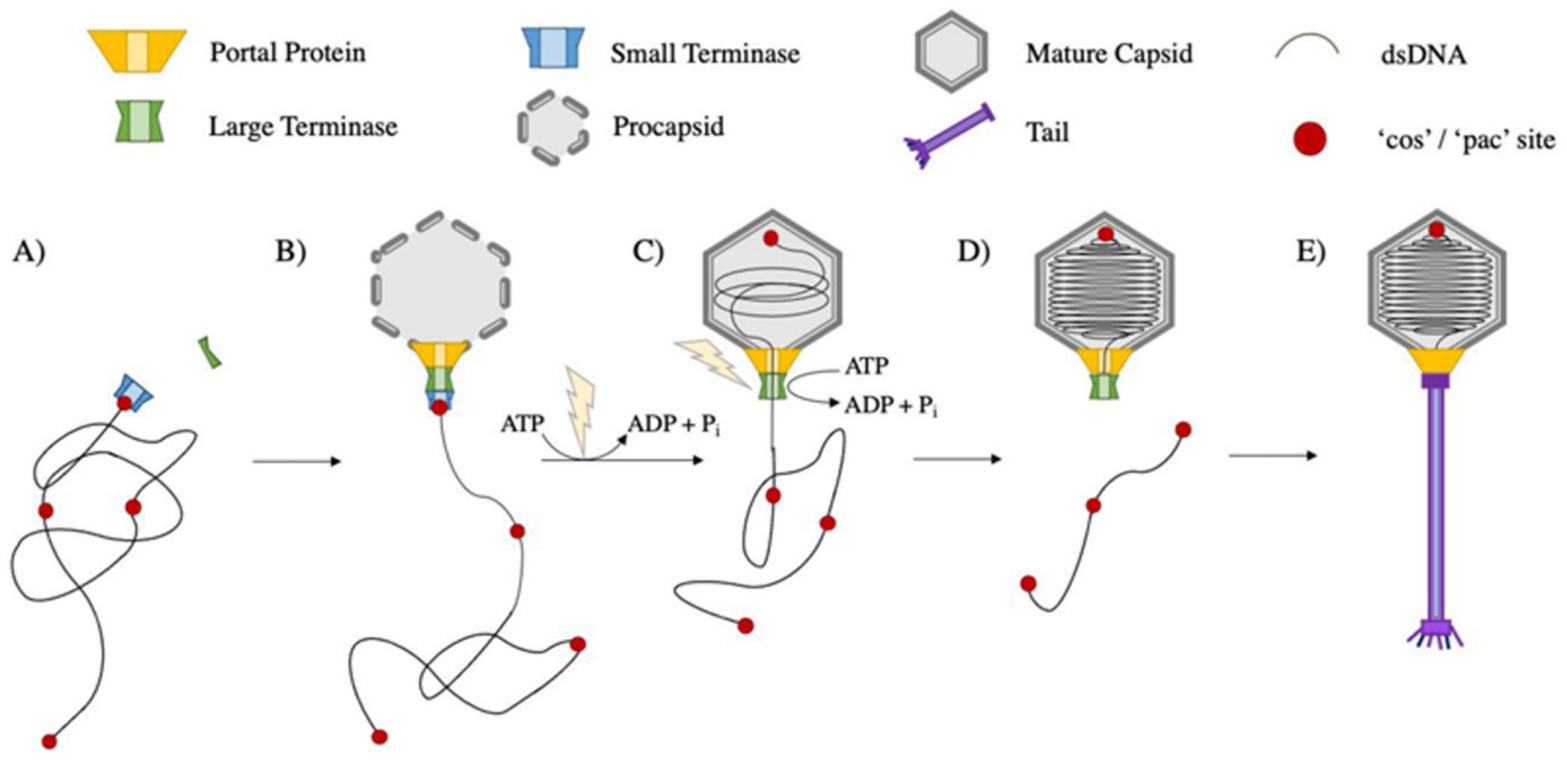
dsDNA bacteriophage assembly. A) Small terminase recruits large terminase to the viral genomic DNA B) Large terminase docks onto the portal vertex of the preformed protein prohead C) Large terminase powers packaging of DNA into the prohead using ATP hydrolysis D) the prohead expands into a mature capsid and DNA is cleaved after one genome length has been packaged E) the terminase complex dissociates and is replaced by neck and tail proteins to form a mature virion

In terminase packaging systems, a dodecameric portal protein at a unique pentameric vertex of the prohead, represents the site for prohead assembly, DNA packaging and later tail attachment. DNA packaging triggers the maturation of proheads into expanded, DNA-filled capsids which show thinner shells, sharper vertices and a larger diameter despite displaying the same T-number and possessing the same number of viral coat proteins (Murialdo and Becker 1978; Earnshaw and Casjens 1980; Tu et al. 2001). Additional proteins often contribute to capsid stability or host recognition. The DNA packaging terminase complex consists of a ring of large terminase subunits (Hendrix 1998) which translocates the genome into the prohead in an ATP dependent manner (Daudén et al. 2013; Migliori et al. 2014; Mao et al. 2016; Dai et al. 2021; Reyes-Aldrete et al. 2021). Small terminase subunits are required for viral DNA recognition, and likely form a second ring beneath. After packaging is completed, the terminase cleaves the DNA, dissociates, and the complex is replaced by neck proteins to prevent the release of genetic material. This is followed by attachment of a pre-assembled tail or tail proteins (Camacho et al. 1979; Rossmann et al. 2004; Lander et al. 2009) to complete virus assembly (Fig. 1).

### Genome packaging strategies

**Figure 2:**
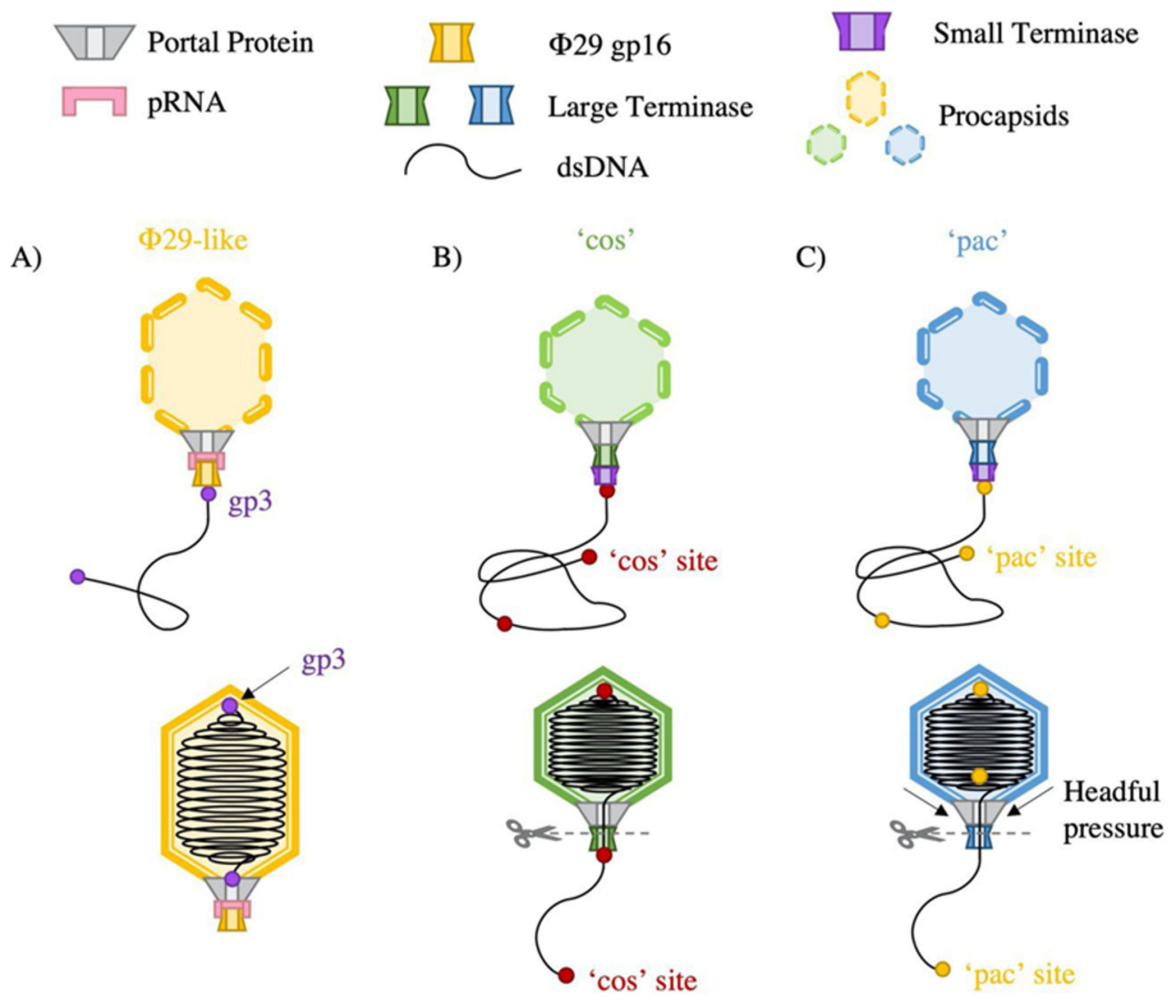
DNA packaging strategies by ds DNA phage A) Φ29 like phages package protein capped genome lengths B) cos phage package cos site capped genome units C) pac phage package in excess of one complete genome length

Although the mechanism of DNA packaging is generally conserved among the tailed dsDNA bacteriophages, further classifications can be highlighted based on processing of the viral DNA (Fig. 2). Most bacteriophage replicate DNA as concatemers containing multiple consecutive copies of the genome (Black 1989). Packaging occurs in a series of unidirectional packaging events along the concatemer, where cleavage of the DNA substrate at termination, produces a fresh dsDNA end for a subsequent initiation event. Packaging series typically consist of two to five full genome packaging events but may elongate depending on conditions (Adams et al. 1983).

Replication of the phage Φ29 DNA unusually produces unit genome length copies, where each 5’ end is covalently bound to viral protein gp3 (Guo et al. 1987). The DNA protein-complex is then packaged by the large terminase motor, here referred to simply as “ATPase”, where a pentameric RNA molecule bridges the large terminase-portal interaction (Fig. 2A)(Reid et al. 1994; Ding et al. 2011). This prohead RNA (pRNA) is necessary for both packaging initiation and substrate selectivity(Zhao et al. 2015b),(Peixuan et al. 1987).

Pac viruses, such as P22, P1, SPP1 and T4, initiate packaging from a specific *pac* (packaging) site within the genome (Tye et al. 1974; Sternberg and Coulby 1990; Tavares et al. 1996; Lin and Black 1998). For SPP1, two separate small terminase oligomers are thought to bind DNA at sequences *pac* L (100 base pairs) and *pac* R (30 base pairs)(Chai et al. 1995). This loops DNA between binding sites, exposing a third sequence, *pac* C, to cleavage by large terminase. Pac virus termination occurs via a ‘head full’ mechanism (Jackson et al. 1978), whereby pressure changes within the capsid are relayed to the terminase. This stimulates large terminase nuclease activity required for DNA cleavage. In practice, a small amount of redundant DNA is always packaged with SPP1 virions harbouring approximately 103% of the genome length (Oliveira et al. 2013) (Fig. 2 C).

*Cos* viruses, such as λ, HK97 and P2, instead package a single genome-length DNA (Ziermann and Calendar 1990; Juhala et al. 2000). Upon bacterial infection, the linear genome is injected into the cell, where complimentary sticky ends are sealed by a host ligase. This forms a circular viral genome, which serves as a template for rolling circle replication where the site of adhesion is known as the *cos* site. This sequence is recognised by small terminase, which recruits large terminase to instigate a staggered, sequence-specific cut, producing complimentary overhangs with either a 5’ or 3’ extension (Feiss and Catalano 2007).

For *λ* phage, the core element within the 200 base pair *cos* sequence is *cos N*, where large terminase produces nicks separated by 12 base pair overhangs (Feiss et al. 1983a, 1983b; Hohn 1983). Downstream element *cos B* is also required for efficient initiation of packaging Click or tap here to enter text.Packaging termination occurs at the next *cos* site, where large terminase once again cleaves the *cos* site and efficient termination is reliant on upstream element *cos* (Fig. 2 B) *Q* (Cue and Feiss 1993). The minimal DNA binding site for HK97 has been pinpointed to 15 to 29 base pairs downstream of the *cos* site, presumably positioning large terminase in place for cleavage (Chechik et al. 2023). Termination for both *λ* and HK97 is also proposed to encompass a headful element, as the efficiency of cleavage is highly dependent on the length of DNA packaged (Feiss et al. 1977; Hawkins et al. 2023).

In common with dsDNA bacteriophage, human *Herpes* virus genome replication also occurs in a rolling concatemeric fashion. DNA sequences *pac* 1 and *pac* 2 must be recognised and cleaved for subsequent packaging of genome length units into preformed proheads (Adelman et al. 2001). The delineation of DNA packaging activities (i.e DNA recognition, cleavage, and ATP powered translocation) does not strictly adhere to small terminase and large terminase assignments. Indeed, for HSV1 a total of 7 gene products have been implicated in DNA packaging (Heming et al. 2017). Most prominently pUL15 demonstrates ATPase activity critical for packaging, in common with large terminases, but adopts a 50% larger structure including additional domains. The pUL15 complex is also coordinated by two extra proteins PUL28 which PUL33 (Yang et al. 2020). Cytomegalovirus proteins pUL56 and pUL89 have each been implicated in packaging. pUL56 displays ATPase activity and also recognises *pac* sites 1 and 2 (Bogner et al. 1998; Heming et al. 2017) while both proteins demonstrate nuclease activity (Scheffczik et al. 2002). Mutational and structural studies of pUL89 have revealed residues critical for nuclease activity and DNA binding (Theiß et al. 2019).

### The portal protein

**Figure 3:**
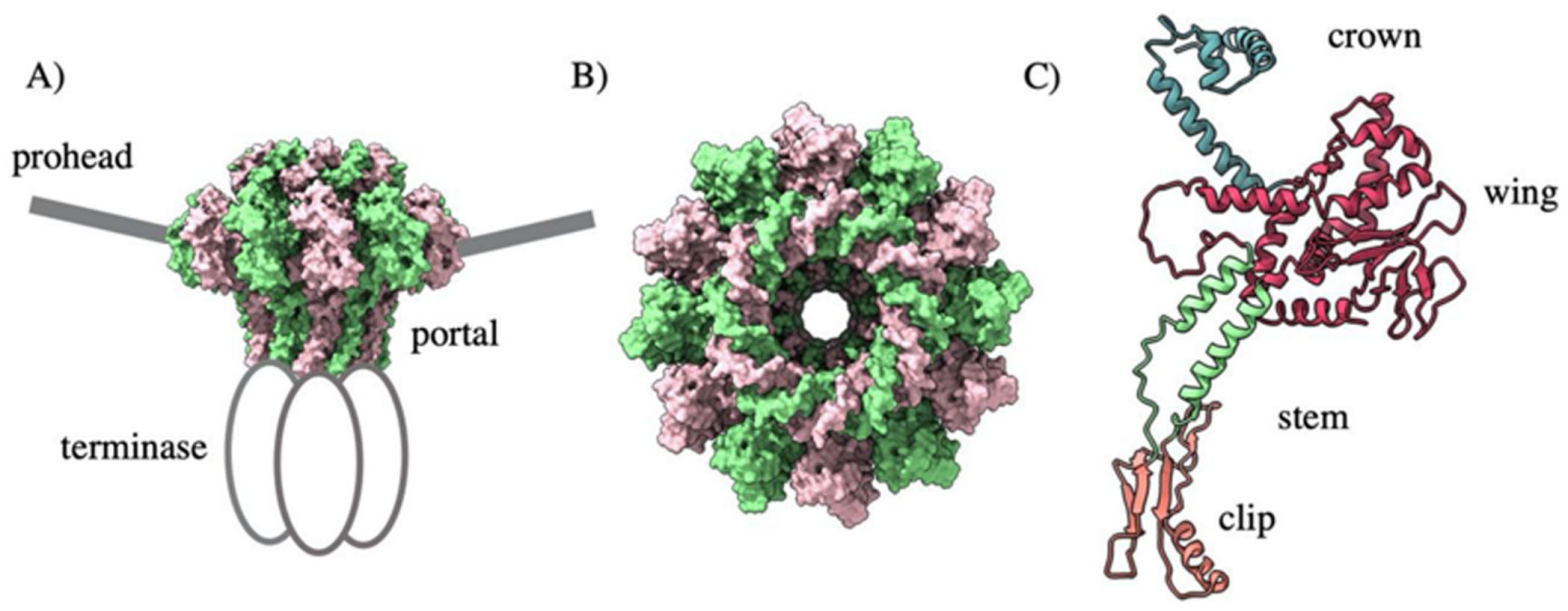
The T4 portal protein BDB 3JA7. A) The portal is positioned at the unique prohead vertex and coordinates binding of the motor B) Portal proteins from dodecameric rings C) The portal protein domain architecture

The portal protein exists at the unique prohead vertex and acts as a door in to the prohead (Fig. 3 A). Despite dramatic divergence of sequence and size, portal proteins always assemble as dodecameric rings (Fig. 3 B) (Valpuesta et al. 1992; Agirrezabala et al. 2005; Doan and Dokland 2007) with a common homology comprised of a clip, stem, wing, and crown domain (Fig. 3 C). The wing domain shows the widest structural variation and facilitates contact with the coat proteins. Meanwhile, the crown interacts with packaged DNA and the stem lines the internal channel, which is negatively charged for the smooth passage of DNA. The clip domain makes contact with the packaging machinery during assembly, and later the adaptor proteins for tail attachment. An additional barrel domain is found above the crown within the portal proteins of many *podoviruses* (Tang et al. 2011). Portal incorporation is critical for native capsid formation in phages T4, SPP1, and Φ29 (Guo et al. 1991; Dröge et al. 2000).

Portal proteins act as interaction hubs throughout the packaging process, coordinating the binding and release of large terminase and later tail proteins. The portal also interacts with the surrounding prohead and remains bound throughout the capsid maturation pathway. Such a variety of binding partners, and the inherent symmetry mismatches of the system, demands plasticity and flexibility of the portal protein. Indeed, asymmetric reconstructions of portal protein structures *in situ* show deviation form C_12_ symmetry. For instance, a recent structure of the PaP3 portal displays a “corkscrew” architecture: employing helical, rather than rotational quaternary structure (Hou et al. 2022). Meanwhile, an asymmetric reconstruction of the T4 unique portal-containing vertex reveals significant structural variation within the portal protein N-terminus. This permits similar interactions with the capsid’s coat protein subunits, which in turn display only minimal conformational deviation from each other (Fang et al. 2020). *In situ* structures of portals in Φ29, P23-45 and P68 show poorly resolved N-terminal whiskers (Bayfield et al. 2019; Hrebík et al. 2019; Xu et al. 2019) which could implicate the N terminus more generally in this symmetry breaking function. The clip domain of the HK97 portal also shows deviation from C_12_ symmetry when in complex with large terminase (Hawkins et al. 2023).

The plasticity of portal proteins is utilised for signal transduction. In *pac* viruses, packaging termination is instigated in response to the increasing internal pressure as DNA fills the capsid. This pressure change is thought to be relayed to the large terminase through the portal protein (Lokareddy et al. 2017). For P22, comparative structures of prohead and mature phage portals reveal an increase in portal density and reduction in portal volume with the progression of DNA packaging. DNA is also spooled around the mature phage portal structure in a configuration that computational modelling deems incompatible with the procapsid portal form (Lokareddy et al. 2017). In corroboration of this suspected signal transducing function, several discrete mutations within the P22 portal core produce an overpacking phenotype, where portals remain in a more ‘procapsid like’ form. This in turn prevents termination (Bedwell and Prevelige 2017). The T7 portal protein also undergoes a striking structural transformation between its procapsid and mature forms. Within the mature head of T7, the portal is compressed outward from the capsid centre, reducing its radial length by 12 Å and exposing the clip domain. This is facilitated by a 25° rigid body rotation of the wing domain (Chen et al. 2020).

The role of the portal as a DNA pressure sensor is similarly apparent in other ds DNA phage which do not employ a headful termination strategy. Mutations within the λ phage portal protein core have been shown to impede termination (Wieczorek et al. 2002). Furthermore, it has been proposed that HK97 relies on portal-mediated pressure changes for the release of its large terminase (Hawkins et al. 2023). Meanwhile, the mature Φ29 portal protein shows a 16 Å reduction of the diameter of the clip domain, and a comparable expansion at the top of the wing domain relative to the procapsid form (Xu et al. 2019).

The portal protein also plays a key role in preventing DNA leakage after packaging has been terminated. P22 mature head portal shows a dramatic decrease in the channel diameter from the procapsid form, from 40 Å to 25 Å (Olia et al. 2011; Lokareddy et al. 2017; Dedeo et al. 2019). This facilitated by a 10° increase in the angle of tunnel helices towards the central axis. A similar phenomenon is apparent for *Thermophage* P23-45, where the *in situ* prohead structure displays a constricted channel relative to the crystal structure (Bayfield et al. 2020). Here, channel loop conformations are inverted, reducing the diameter to just 14 Å and subsequently altering the nature of the channel interface from hydrophilic to hydrophobic. This is accompanied by positional adjustment of the crown domain (Bayfield et al. 2020). In addition to these broad structural rearrangements, portal proteins also display mechanisms to prevent DNA slippage during packaging. Pairs of charged residues within the clip region (K200 and K209 for Φ29, and K331 with K342 for SPP1) (Chaban et al. 2015) are thought to act as a DNA clamp. Single particle studies on T4 also indicate that the portal prevents DNA release during motor slipping or stalling (Fuller et al. 2007a).

### The small terminase

Most dsDNA phage employ small terminase proteins for recognition of the viral genome. The structure of numerous small terminases have been determined to reveal a central oligomerisation domain joining the N-terminal DNA-binding domain and C-terminal large terminase binding domain (Hamada et al. 1986a; Hamanda et al. 1986b; Casjens et al. 1987; Rao and Black 1988). Whether small terminase remains bound to large terminase throughout the packaging process remains unclear.

**Figure 4:**
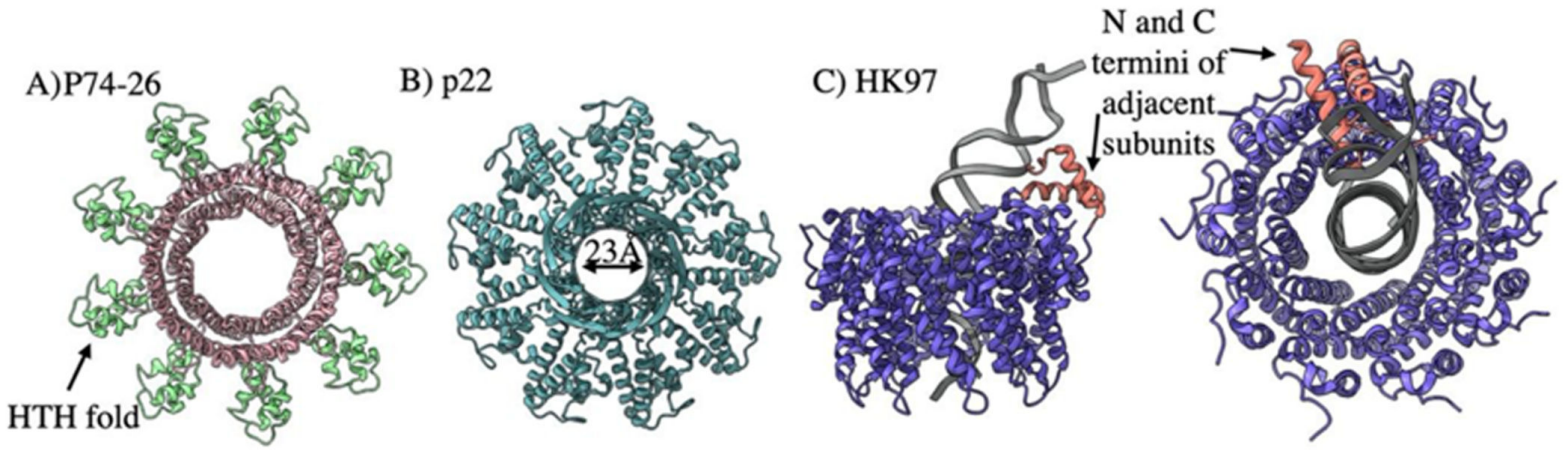
Small terminase architecture A) P74-26 small terminase. PDB 6V1I B) p22 small terminase. PDB 3P9A C) The HK97 complex in complex with DNA. Two unfolded regions fold into helices on DNA binding. PDB 8POP

Historically, the nature of the small terminase DNA interaction at packaging initiation has proved a subject of debate. One school of thought favours the passage of DNA through the small terminase central channel, while another predicts DNA wrapping around the outside of the oligomeric ring. SPR and EPR data for Sf6 small terminase support this wrapping hypothesis, as binding between small terminase and DNA is shown to be weak but cooperative (Büttner et al. 2012). Indeed, this small terminase can bind the genome of related phage SPP1 and so specificity is thus thought to be determined more by DNA shape (i.e intrinsically bent) rather than sequence. This is supported by the structures of p74-26 (Fig. 4 A) and T4 like small terminase oligomers (Suna et al. 2012) which each show DNA binding HTH motifs arranged radially (Hayes et al. 2020). However, it has been argued that such bending of DNA is energetically unfavourable and a high-resolution structure of the P22 small terminase oligomeric ring features a sufficiently wide lumen for hydrated B -DNA (Fig. 4 B) (Roy et al. 2011).

Recently, the first structure for a small terminase bound to a DNA substrate has been determined (Chechik et al. 2023). Here, the HK97 small terminase shows a nonameric ring encircling DNA, with the disordered N- and C-terminus of two adjacent subunits folded into helices which form a DNA binding substructure (Fig. 4 C). Two separate arginine residues, which are positioned within the major and minor DNA grooves, appear to stabilise the DNA in a bent conformation. The small terminase is thus locked in place in a sequence specific manner, presumably allowing the ring structure to slide along the DNA substrate freely until it reaches the binding site. This is supported by the recent model of λ small terminase as a “sliding clamp”, limiting back slipping of large terminase (Rawson et al. 2023). While there is wide variation amongst other small terminase oligomeric states and reported structures, the N-terminus is rarely well resolved and always includes at least one positively charged residue. This suggests that folding of unstructured regions in response to specific DNA binding may be conserved. However, it is of note that the isolated DNA binding domain of some small terminases can be expressed, while others are entirely unknown, and that some dsDNA phage do not employ small terminases at all.

### The large terminase

**Figure 5:**
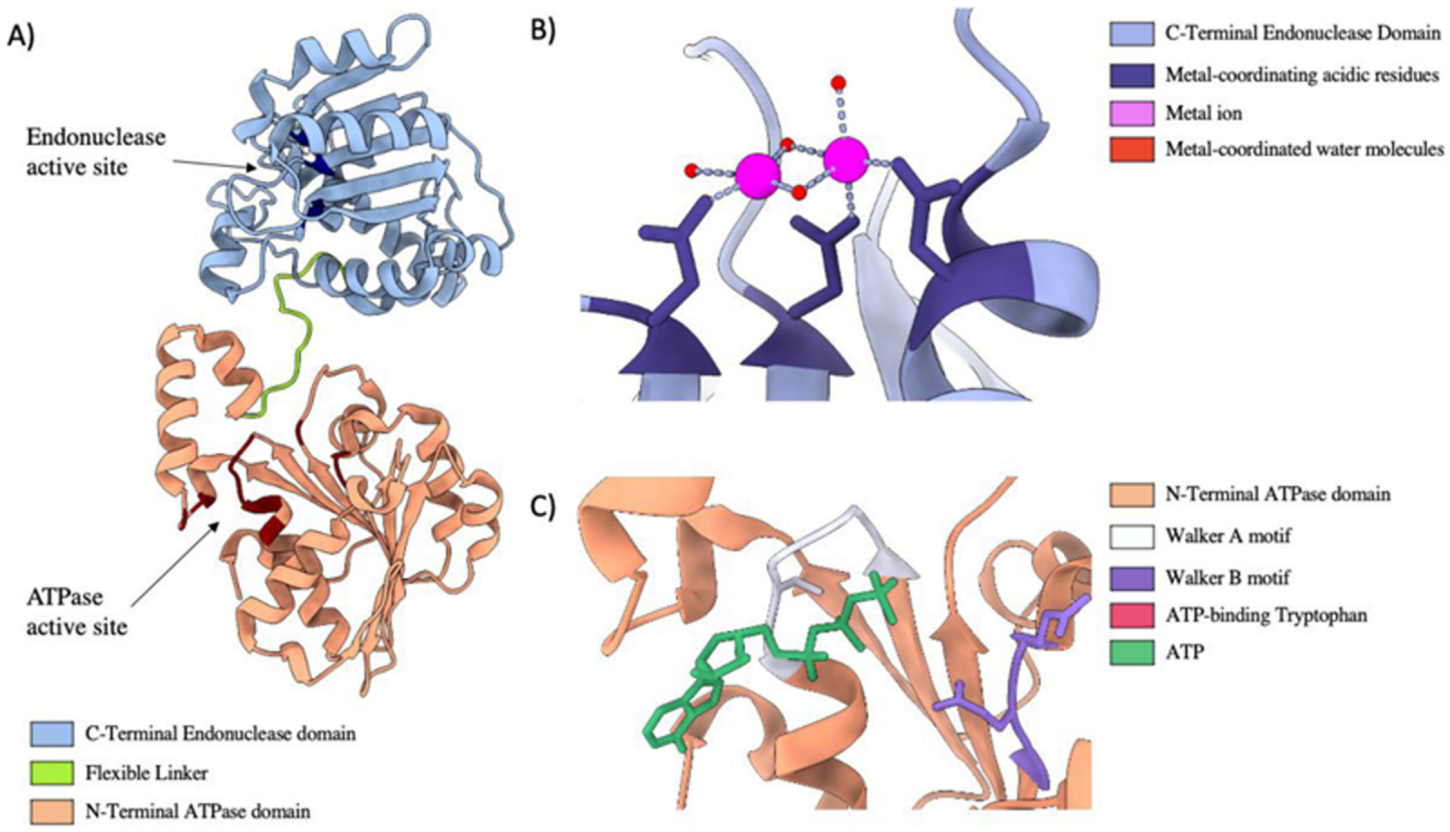
Large Terminase Structure A) Ribbon diagram of bacteriophage Sf6 large terminase, PDB code 4IFE. B) The nuclease active site of bacteriophage SF6, coordinating Manganese ions, PDB code 5C15. C) The ATPase active site of the Sf6 large terminase coordinating ATP, PDB code 4IFE

Translocation of DNA into the capsid is powered by the large terminase consisting of an N-terminal ATPase domain (Morita et al. 1993) adjoined to a C-terminal endonuclease domain by a flexible linker (Fig. 5). The terminase functions akin to other ring-shaped, oligomeric translocases: utilising ATP hydrolysis cycles to translocate biological monomers through a central pore (Lyubimov et al. 2011). Once a complete genome is packaged the large terminase nuclease domain is responsible for DNA cleavage.

Terminases belong to the additional strand, conserved glutamate (ASCE) subset of P-loop NTPases, which employ conserved Walker A and B motifs for ATP binding and hydrolysis (Iyer et al. 2004a; Ogura et al. 2004; delToro et al. 2019). The ASCE family utilise a second conserved acidic residue within the Walker B domain, and a β-strand inserted between the Walker A and B domains (Leipe et al. 2003; Iyer et al. 2004b). β and γ phosphates of ATP are coordinated by the Walker A domain, while the Walker B motifs coordinates Mg^2+^ (Walker et al. 1982). This facilitates the conserved catalytic carboxylate to activate water for ATP hydrolysis, which in turn instigates conformational change to translocate the substrate (Kenniston et al. 2003; Hanson and Whiteheart 2005). Each hydrolysis event instigates a subsequent ATP hydrolysis event in the neighbouring subunit (Moffitt et al. 2009; Mao et al. 2016).

The C-terminal domain of large terminases strongly resemble each other (Sun et al. 2008; Smits et al. 2009; Nadal et al. 2010; Roy and Cingolani 2012; Zhao et al. 2015a; Xu et al. 2017b), adopting the RNase H-like fold and mechanism. This fold is shared by the C-terminus domain of HSV1 packaging protein UL15 (Adelman et al. 2001) and CMCV packaging protein UL89 (Nadal et al. 2010). The nuclease domain adopts a two-metal catalysis mechanism where each metal is coordinated by active site carboxylate groups (Nowotny et al. 2005). Metal A activates a coordinated water for nucleophilic attack, and metal B stabilises the transition state oxyanion leaving group. The structure of the *Thermus thermophilus* bacteriophage G20C nuclease domain indicates a Ruv-C type mechanism which requires Mn^2+^, Mg^2+^ or Co^2^ for functionality (Xu et al. 2017b). While this mechanism is widely considered to be adopted by all large terminase, two structures have inspired conflicting ideas. The Sf6 nuclease structure suggests the two metals ions are bought unusually close (2.42 Å) during catalysis (Zhao et al. 2015a). In stark contrast, the two magnesium ions identified in the recent *Pseudomonas* phage E217 large terminase structure, appear overly separated for two metal catalysis. This suggests rearrangement of the active site is necessary for nuclease activity (Lokareddy et al. 2022).

### Translocation kinetics of the large terminase packaging motor

The large terminase packaging motor inspires particular mechanistic intrigue, as a result of the extreme repulsive forces it must overcome. Within the prohead, DNA is packaged to a crystalline density, which represents a highly unfavourable energetic state. Not only must the electrostatic repulsion of confining the negatively charged DNA molecule be accounted for, but also the necessity for DNA bending and dehydration (Riemer and Bloomfield 1978; Purohit et al. 2005; Petrov and Harvey 2008). It is thus perhaps unsurprising that viral terminases represent the most powerful biological machines studied, reaching forces of up to 100 pN (Chemla et al. 2005; Fuller et al. 2007b; Rickgauer et al. 2008). In addition, the motor must remain stable throughout packaging, in spite of the symmetry mismatch between the portal and large terminase oligomers.

Much mechanistic understanding of viral DNA packaging motors has been drawn from single particle optical tweezer studies. Here, a DNA substrate is tethered to a micro bead held in place by a laser beam (Ashkin et al. 1986; Fuller et al. 2006; Moffitt et al. 2008). When a small external force is applied, i.e when the DNA is packaged, the force can be measured by the laser beam which applies an equal force in the opposite direction.

**Figure 6:**
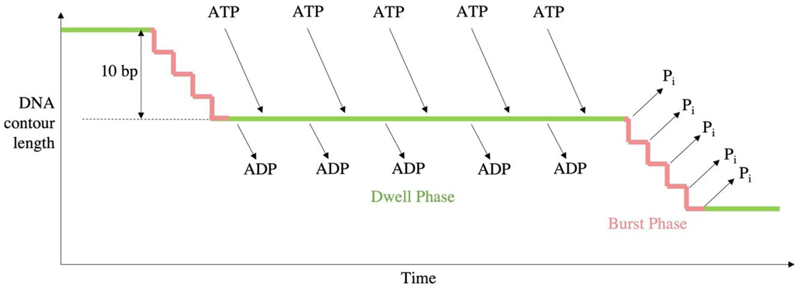
Kinetic cycle of Φ29. Timeline of the burst/dwell cycle, adapted from (Chistol et al. 2012). Five sequential ATP hydrolysis events produce just four translocation steps of 2.5 bp, followed by sequential ADP release and ATP binding.

Single particle studies of the Φ29 motor indicated that packaging proceeds through two alternating modes: the dwell phase and burst phase. During the dwell phase ATP binds cooperatively to each subunit around the ATPase ring. During the burst phase, DNA translocation into the prohead occurs in 4 subsequent 2.5 bp steps corresponding to 4 ATP hydrolysis events (Fig. 6) (Moffitt et al. 2009). As packaging of the genome approaches completion, pausing and slipping of the motor occurs more frequently and the burst phase step size decreases to approximately 2.3 bp per hydrolysis event, whilst dwells lengthen (Liu et al. 2014).

Single molecule studies have also been used to probe T4 and *λ* DNA packaging machines. For each phage, the rate of translocation, reaching an average of 700 bp/s for T4 (Fuller et al. 2007a), is proportional to the size of the genome. This means the total genome packaging time corresponds to roughly 2-3 minutes for each. Reduced packaging velocity, as internal pressure increased towards packaging completion, was also ubiquitous. However, in the case of bacteriophage *λ*, an early pressure peak followed by a drop was also observed after approximately 30 % of the genome had been packaged. This pressure is considered responsible for prohead expansion, where the mature capsid displays a twofold increase in volume, relieving some internal pressure (Fuller et al. 2007b). For T4, packaging appears especially versatile, with reduced slipping and packaging into a mature capsid occurring with equal velocity to the immature prohead (Zhang et al. 2011). Single molecule fluorescence studies have also revealed that multiple DNA substrates may be packaged into a single prohead in sequential packaging events (Vafabakhsh et al. 2014).

### Coordination of the DNA motor

Structural events within the motor require tight coordinated in order to package DNA efficiently into the prohead. The first point of regulation is often the small terminase, which selectively recruits viral DNA to the large terminase motor, in preference to host DNA. Next, cycles of ATP hydrolysis by large terminase subunits must be coupled to DNA translocation. This requires not only intra-subunit coordination, but also inter-subunit communication for the motor to function as a single entity. Lastly, timely termination of packaging requires strict regulation of the large terminase endonuclease domain. Endonuclease activity must remain inactive throughout packaging and be stimulated only after a full genome length has been packaged.

Small terminases have been shown to stimulate large terminase packaging activity (Al-Zahrani et al. 2009). For instance, binding of homologous T4 small terminase (gp16) to large terminase (gp17) is proposed to stimulate a conformational change which repositions residues within the large terminase ATPase catalytic pocket (Baumann and Black 2003; Al-Zahrani et al. 2009). Numerous sites of interaction between the two proteins have been identified, suggesting that multiple weak interactions could facilitate the rapid multimeric assembly and disassembly required for packaging. For bacteriophage P22, it has been deemed critical for small terminase to its cognate viral DNA in order to stimulate large terminase. This depicts an elegant way of discriminating against wasteful packaging of host DNA (Roy et al. 2012). Interestingly, the bacteriophage P74-26 small terminase has been shown to stimulate the ATPase activity of large terminase, while simultaneously inhibiting the endonuclease activity (Hayes et al. 2020), presumably preventing the motor from premature termination.

Coordination of the ATP active site and DNA binding site within the large terminase monomer has largely been informed by crystallography. ATP bound large terminase subunits are broadly thought to adopt a ‘closed’ or ‘tense’ conformation with high DNA affinity. Hydrolysis of ATP causes movement between the two domains, coordinated by the linker or lid domain, as well as rearrangement of the DNA binding site. Now, in the ‘open’ conformation, large terminase releases DNA, facilitating its passage into the prohead. This model is supported by comparative crystal structures of monomeric terminases in apo, ADP-bound, and ATP analogue-bound conformations. In particular, significant conformational changes have been observed between ATP analogue-bound and apo structures of the P74-26 large terminase, where an approximate 13° rotation occurs between the ATPase and lid subdomains.

Structures of T4, P74-26 and Sf6 large terminase monomers each reveal an additional conserved arginine in the Walker A motif. Molecular dynamics simulations indicate that this arginine may act as a ‘toggle’, switching coordination from the active site to a glutamate within the ATPase lid (or linker domain) on substrate release (Ortiz et al. 2019). A crystal structure of the Sf6 large terminase with bound ATPγS shows critical Arginine residue R24 coordinating the gamma phosphate. On ATP hydrolysis and Pi release, R24 instead interacts with and E187 in the lid domain, as shown in the ADP bound structure. This conformational change is proposed to be propagated to the bound DNA, which is subsequently ‘pushed’ into the prohead (Zhao et al. 2013).

In addition to this conserved ‘sensor’ arginine, MD simulations have indicated a single glutamate switch residue as critical to the transition between the ATP bound DNA tight binding state, and the ADP bound state. In each of the four large terminases investigated, Sf6, φ29, ascc-φ28 and P74-26, a polar or charged switch residue appears to “fix” the catalytic glutamate to point away from the active site rendering it inactive. The switch residue in turn relays structural rearrangement at the DNA binding site to promote binding when in the presence of ATP (Pajak et al. 2021a).

**Figure 7:**
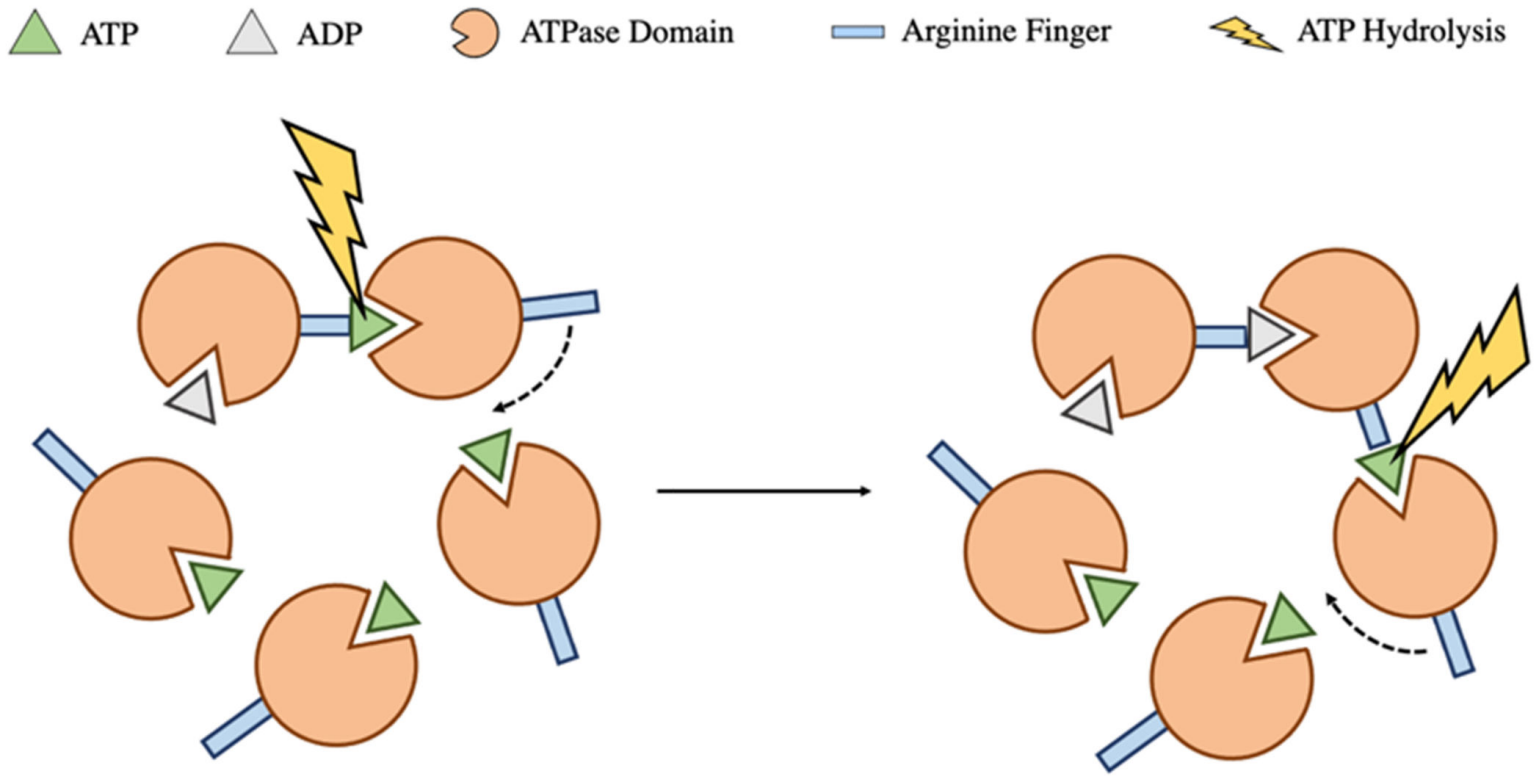
Coordination of ATP hydrolysis. On ATP hydrolysis, conformational change within a single large terminase subunit repositions the trans acting arginine finger into the adjacent ATP active site catalysing a subsequent hydrolysis event. This occurs sequentially around the ring.

Subunit cooperativity is also critical for the motor function, with purified large terminase monomers displaying limited spontaneous ATPase activity, incompatible with demands of DNA packaging (Tafoya et al. 2018). The additional stimulation is thought to be provided by a trans-acting arginine finger, from a neighbouring subunit, which coordinates the ATP γ phosphate (Fig. 7). Trans acting arginine fingers have been identified for Φ29, P74-26 and D6E, each of which is indispensable for ATP hydrolysis (Mao et al. 2016; Hilbert et al. 2017; Xu et al. 2017a). The conformational change associated with ATP hydrolysis in one subunit, is proposed to insert this residue into the active site of the adjacent subunit, producing a burst of sequential ATP hydrolysis events around the ring as catalogued by the Φ29 dwell burst translocation cycle. It is of note that, for Φ29 at least, the first hydrolysis event must occur without a trigger, and also that only 4 of these 5 hydrolysis events produce translocation steps, indicating a unique role for a single subunit (Mao et al. 2016). The D6E arginine finger position is conserved among the FtsK/HerA superfamily, where the same mechanism is widely employed for coordination (Xu et al. 2017a). Unusually, extensive mutational studies on HK97 large terminase, in conjunction with the high-resolution crystal structure and a functional packaging assay, eliminate all potential trans acting arginine finger candidates. Instead K57 is proposed to act in cis, providing the additional charge for ATP coordination (Fung et al. 2022).

Regulation of the large terminase nuclease domain is critical during packaging to prevent early termination. Stimulation is thought to be modulated by the N-terminal ATPase domain. Indeed, in the absence of the ATPase domain, the isolated P22 endonuclease domain shows reduced or obliterated activity. An additional extended β-sheet and an auxiliary β-hairpin are seen in terminase nuclease families relative to the classical RNaseH type fold. Since this β-hairpin is positioned to block dsDNA access to the nuclease active site, it has been proposed to play the role of the nuclease activity “switch” (Smits et al. 2009; Roy and Cingolani 2012). For *cos* viruses such as HK97, small terminase is expected to play a role in positioning large terminase for DNA cleavage (Hawkins et al. 2023; Chechik et al. 2023). In *pac* viruses, however, the headful pressure from within the prohead is likely propagated through the portal protein to stimulate packaging termination, as discussed earlier.

### Models for DNA translocation from cryo-EM structures

**Figure 8:**
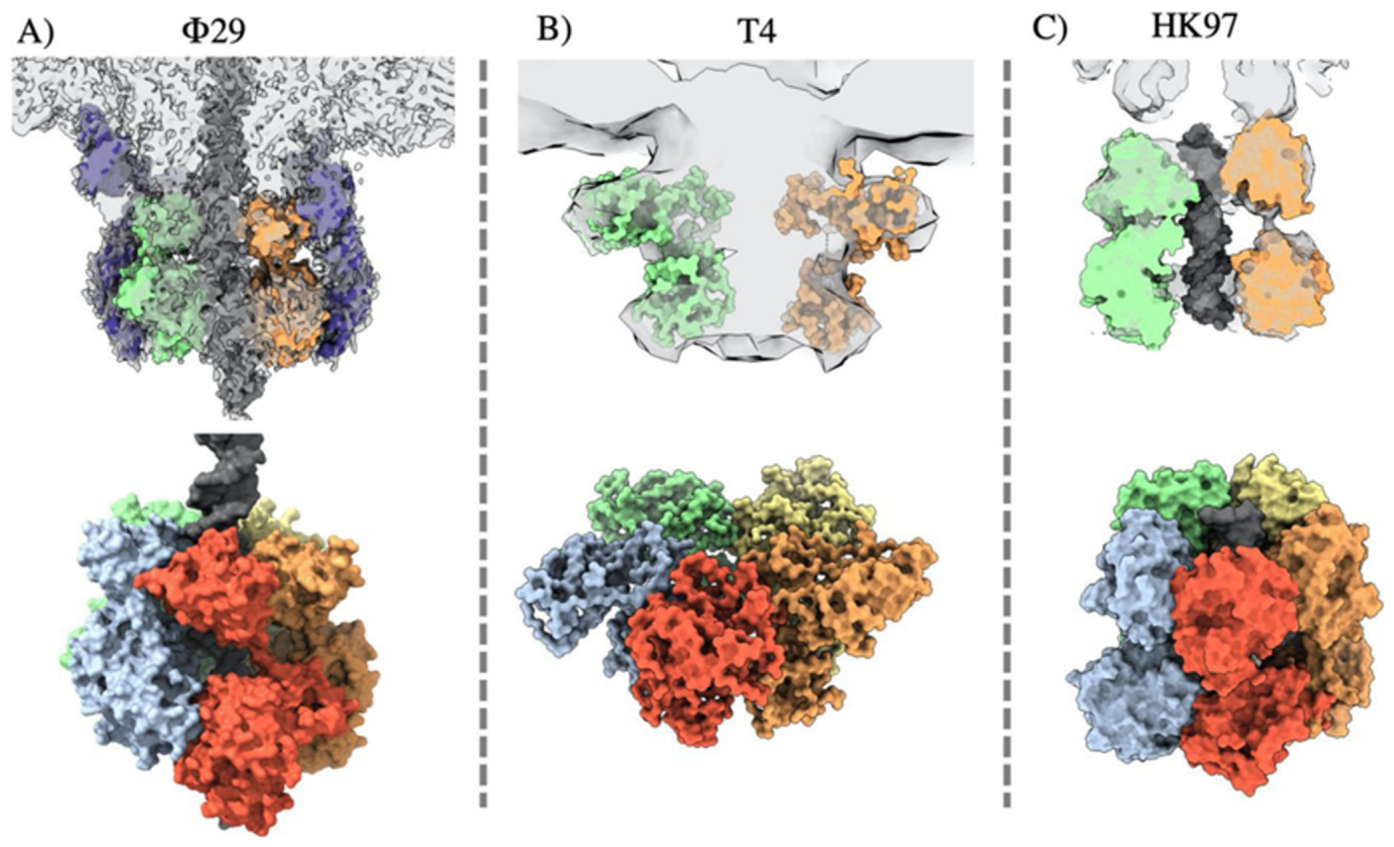
Comparison of complete packaging complexes of ds DNA phage. Cryo-EM reconstructions are shown for: A) Φ29 EMDB 22441 fitted PDB 7JQQ, B) T4 EMDB 1572 fitted PDB 3EZK, C) HK97 EMDB 16653 fitted PDB 6Z6D

Over the last decade, several structures of complete dsDNA viral packaging systems have been resolved by cryo-electron microscopy, in spite of the inherent challenges posed by the flexibility, asymmetry, and transient association of these complexes. These reconstructions include active packaging systems for Φ29, T4 and HK97 (Fig. 8), and isolated packaging motors, in the absence of DNA or proheads, for T7 and HSV1. Such structures, in combination with the wealth of complimentary data from single particle studies, molecular dynamics simulations, and structures of individual protein constituents, have informed new understanding of DNA packaging mechanisms.

A high resolution cryo-EM reconstruction of the intact Φ29 packaging motor, comprising the capsid, pRNA, ATPase and DNA, shows the five large terminase ATPases arranged in a “cracked” helical conformation, stabilised by ATPγS (Fig. 8 A) (Woodson et al. 2021). This contrasts previously determined “planar” assemblies of the Φ29 ATPase (Koti et al. 2008; Morais et al. 2008), as well as an ADP bound planar structure of the highly related phage ascc-φ28 large terminase (Pajak et al. 2021b). The necessary transition required between these two states has been modelled by molecular dynamics simulations and inspired a translocation model that agrees with the burst-dwell cycle (Pajak et al. 2021b; Woodson et al. 2021)

**Figure 9:**
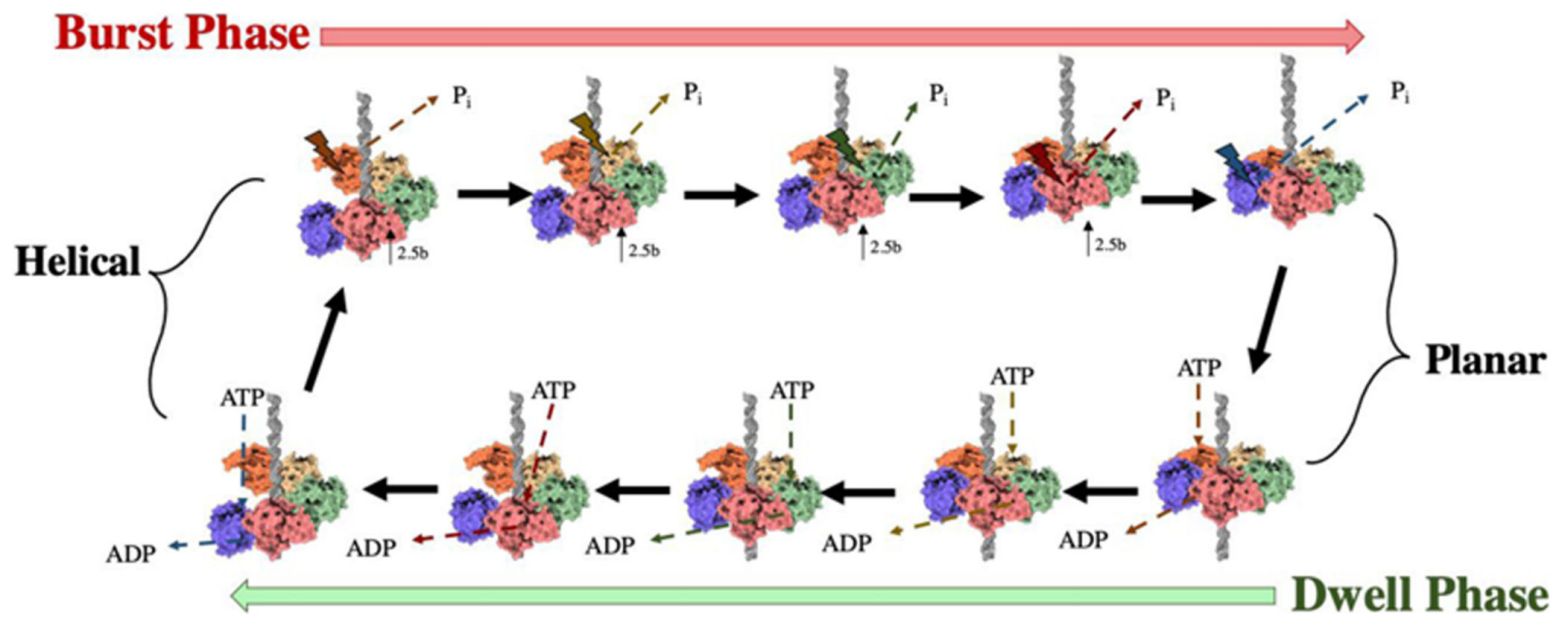
Schematic of the Φ29 packaging mechanism. The ATPase domains of the large terminase pentameric ring sequentially transition from a cracked helix to planar arrangement in agreement with the dwell- burst kinetic cycle

In the ATP-bound cracked helical conformation, all subunits tightly grip the DNA. When Subunit 1 hydrolyses ATP it releases DNA, and Subunit 2 is moved up into the Subunit 1 plane, moving approximately 2.5 bp of DNA into the prohead. Subunit 1 residue K105 is now poised to trigger ATP hydrolysis in Subunit 2, which in turn loses grip of the DNA and brings Subunit 3 into the Subunit 1 plane. Thus, four ATP hydrolysis steps occur, resulting in four 2.5 bp DNA translocation steps. When all five subunits are present in a planar ring, the pentamer has twice the buried surface area between subunits as the cracked helical state, and thus represents a more stable state. Subunit 5 must then hydrolyse ATP to release the DNA substrate and prime S1 for nucleotide exchange and the beginning of the dwell phase. Here, ADP-ATP exchange again occurs sequentially, allowing each subunit in turn to move down the helix and make contact with the DNA (Pajak et al. 2021b; Woodson et al. 2021). This model is summarised in Fig. 9. DNA binding is energetically favourable, compensating for the less stable protein conformation, with decreased interaction interfaces between subunits. ATP hydrolysis is thus required to climb out of the energy minima in the transition to the planar state once again (Woodson et al. 2021).

Movement between the ATPase and nuclease domains is coordinated by a linker, folded into a 3-helix bundle, which also makes contact with the adjacent subunit. Variations in twist and pitch of the helices allow subunits to adopt the different orientations required for translocation. The ATPase active site exists at the subunit periphery, where ATP appears to be sandwiched between R146 and K105 from the neighbouring (trans acting) subunit. Whilst all five subunits appear to contact DNA, K56 in S2 - S5 is positioned to the track the 5’ – 3’ DNA strand. Meanwhile in S1, K56 is closer to the 3’-5’ strand, perhaps helping to distinguish a unique role for this subunit in initiating each burst cycle (Woodson et al. 2021).

For the bacteriophage T4, a crystal structure of the apo form of the large terminase and cryo-EM structure of a fully assembled packaging motor have also been derived (Fig. 8 B) (Sun et al. 2008).The monomer depicts a “tense”, more compact state relative to a “relaxed” extended state seen in the low resolution cryo-EM reconstruction. The nuclease domain is thought to bind DNA in the relaxed state, moving it towards the ATPase domain. This was proposed to induce a conformational change which flips an arginine finger from the same subunit (cis) into the active site, catalysing ATP hydrolysis. In turn the nuclease domain rotates aligning charge pairs producing a 2 bp movement of DNA via electrostatic attraction. This represents the tense state. Release of ATP hydrolysis products triggers the relaxation of the large terminase and positions DNA ready to bind the next subunit, so that at any point just one of the five subunits adopts the tense state (Suna et al. 2012).

In place of the burst- dwell sequence adopted by the Φ29 motor, the T4 motor is proposed to operate in a continuous burst cycle. In this model ATP hydrolysis in one large terminase subunit could occur simultaneously to ATP loading in the adjacent subunit, thus removing the ‘dwell’ phase and facilitating faster packaging (Rao et al. 2023). This could account for the 8-fold increased speed of the T4 motor relative to Φ29 (Zhang et al. 2011). A more flexibly coordinated motor may also be responsible for the frequent slipping and restarting of the T4 motor during single molecule studies and its ability to tolerate up to 3 inactive subunits (Kottadiel et al. 2012). Such a mechanism is also supported by enhanced DNA grippingcapacity, and prevention of slipping of ATP bound subunits (Ordyan et al. 2018).

More recently a cryo-EM structure of the HK97 packaging motor has been determined in a pseudo termination state (Hawkins et al. 2023). The motor assembly is highly asymmetric, showing a ring of 5 large terminase monomers tilted against the portal axis at 10°. DNA runs through the channel and appears cleaved at the portal entrance in agreement with packaging termination. Interestingly, new biophysical and structural data for λ phage terminase instead suggests that DNA cleavage occurs in a tetrameric conformation, which welcomes in an additional subunit for packaging (Prokhorov et al. 2022).

Docking five monomers of HK97 large terminase (Fung et al. 2022) reveals variable degrees of extension between N- and C-terminal domains of individual subunits. More extended subunits make clear contact with DNA via both domains, as well as the portal clip domain, and are assumed ATP bound. Meanwhile more compact subunits only make contact with DNA via the C-terminal domain and do not appear portal bound. This suggests ATP powered large terminase contraction may mediate portal contact in addition to DNA translocation. Such asymmetry also likely requires strict coordination between subunits, but interestingly the assembly does not adopt the cracked helix conformation of its Φ29 counterpart.

Two high resolution structures of the HSV1 ds DNA packaging motor have also been determined by cryo-EM, depicting a ring of the large terminase homologue pUL15 subunits in i) an apo and ii) an ATP analogue bound conformation (Yang et al. 2020). Regulatory proteins pUL28 and pUL33, which have no bacteriophage homologs, also feature in the structure, with each of the three proteins interdigitating into one unit of the compact hexameric conformation (Yang et al. 2020). The pUL15 endonuclease domain is positioned away from the centre, suggesting that headful packaging pressure must induce domain rearrangement for the nuclease to engage the DNA at packaging termination.

The hexameric conformation presented represents the major form when the three proteins are co-expressed, and also mimics the oligomeric state favoured by other DNA translocases. This produces a central pUL15 ATPase channel, lined with conserved basic amino acids, whose diameter (39 Å) near matches the portal lumen (36 Å) (Wang et al. 2020). However, it is of note that these structures do not include the viral DNA or capsid, and that a pentameric structure can theoretically be arranged from the pUL33-pUL28-pUL15 unit in which the central channel diameter varies between 19 Å and 24 Å. In addition, essential packaging accessory proteins for both Epstein–Barr virus and Kaposi’s sarcoma-associated herpesvirus each self-associate into pentameric rings with positively charged DNA binding central channels. This implicates association with pentameric terminase machinery (Didychuk et al. 2021).

Despite variations, each of the dsDNA packaging structures show common features which may prevail throughout dsDNA packaging machines. In each system, DNA propagation occurs via contraction of DNA bound subunits, with cycling DNA binding and releasing coordinated by the ATP hydrolysis cycle. This is in fitting with other translocases such as the FtsK and TraB (Jing et al. 2016). For Φ29 and HK97 the large terminase nuclease domain is adjacent to the portal, although for Φ29 this interaction is mediated by pRNA. This orientation is also supported by a low resolution cryo-EM structure of the T7 large terminase-portal complex (Daudén et al. 2013), and FRET data on the T4 packaging motor (Dixit et al. 2013), although the orientation assigned by the low resolution cryo-EM structure was opposing (Sun et al. 2008). As more, and higher resolution structures undoubtably emerge it will be of great interest as to the extent to which DNA packaging machines are conserved between phage and eukaryotic double stranded DNA viruses.

## Conclusion

In this chapter, we catalogue the current understanding of the molecular mechanisms of terminase-based DNA packaging systems of dsDNA viruses. Each utilises a portal protein, which exhibits conformational differences between prohead and mature capsid forms, with deviations from the C_12_ symmetry. This demonstrates the portal protein’s plasticity, which is crucial for interactions with multiple binding partners and for propagation of the ‘headful’ pressure signal from within the prohead to large terminase. Portal protein also acts as gatekeeper to prevent DNA leakage before tail attachment. The mechanism by which small terminase recognises viral DNA and regulates large terminase remains more elusive. However, the recent cryo-EM structure of HK97 small terminase has shed light on a novel DNA binding mechanism involving the folding of disordered protein regions on binding to the *cos* site. It is of great interest weather this mechanism is conserved among other *cos* phage and equally if it translates to *pac* viruses.

For large terminase too, cryo-EM structures have begun to tie together a wealth of data from crystallography, molecular dynamics and single particle studies. Five subunits encircle the DNA substrate, and coordination around the ATPase ring often occurs via a trans acting arginine finger which coordinates the neighbouring active site. For Φ29, a high resolution cryo-EM structure, in conjunction with the dwell-burst kinetic cycle derived from single-molecule data, has inspired a mechano-chemical translocation mechanism, describing sequential conformational changes in each ATPase subunit. Together these result in the transition of the pentameric ATPase from a helical to planar configuration and the propagation of DNA into the prohead. While lower resolution data from other HK97 and T4 packaging motors do not strictly support this model, they each support a subunit contraction driven model. In this way, DNA tethered to an extended ATP-coordinated subunit is propelled into the prohead on ATP hydrolysis, and subsequently released, allowing the process to iterate within an adjacent subunit. This mechanism aligns with other translocases such as FtsK and TraB.

### Grant Support

Wellcome Trust grants 206377, 224665.
